# Editorial: Cognitive impairment and physical function in older adults

**DOI:** 10.3389/fphys.2025.1645677

**Published:** 2025-07-01

**Authors:** José D. Jiménez-García, Daniel Velázquez-Díaz, Antonio Martínez-Amat, Francisco Álvarez-Salvago, Richard B. Kreider, Diego A. Bonilla

**Affiliations:** ^1^ Department of Health Sciences, Faculty of Health Sciences, University of Jaén, Jaén, Spain; ^2^ Translational Research Institute, AdventHealth, Orlando, FL, United States; ^3^ Research Group on Special Populations (FIBIO), Faculty of Biomedical and Health Sciences, Universidad Europea de Valencia, Valencia, Spain; ^4^ Exercise and Sport Nutrition Laboratory, Human Clinical Research Facility, Texas A&M University, College Station, TX, United States; ^5^ Research Division, Dynamical Business and Science Society–DBSS International SAS, Bogotá, Colombia; ^6^ Research Group in Physical Activity, Sports and Health Sciences (GICAFS), Universidad de Córdoba, Montería, Colombia; ^7^ Hologenomiks Research Group, Department of Genetics, Physical Anthropology and Animal Physiology, University of the Basque Country (UPV/EHU), Leioa, Spain

**Keywords:** exercise programs, elderly, cognition, cognitive impairment, physical exercise, aging, SDG3, good health and wellbeing

## Introduction

**FIGURE 1:**
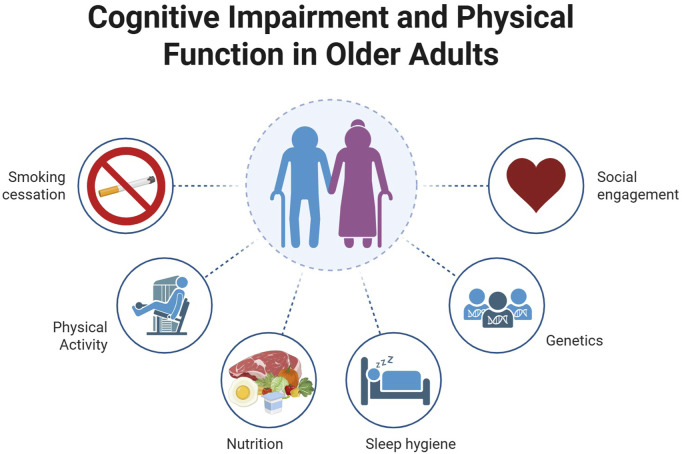
Factors influencing cognitive impairment and physical function in older adults. Health practitioners and professionals should prioritize modifiable lifestyle interventions—particularly physical activity, nutrition, and sleep (The Healthy Trinity) as well as social engagement, and smoking cessation—as cost-effective strategies to delay cognitive/functional decline and reduce mortality in older adults.

The world’s population is growing older, bringing new and complex challenges for public health systems. This editorial synthesizes published articles in the research topic “Cognitive Impairment and Physical Function in Older Adults” which contribute to the advancement in the interplay between modifiable lifestyle factors, social determinants, and cognitive trajectories in older adults. By integrating longitudinal and cross-sectional evidence, we identify key areas for future interventions and emphasize the importance of personalized approaches to support healthy aging in public health (Figure 1).

## Physical activity as a cornerstone of cognitive and physical health

Regular physical exercise demonstrates robust benefits for physical and cognitive health in aging populations (Singh et al., 2025; Izquierdo et al., 2025). A longitudinal study of 10,691 Chinese older adults by Xu et al. revealed that sustained physical activity significantly improved instrumental activities of daily living (IADLs) and reduced depressive symptoms over 8 years, even after adjusting for sociodemographic confounders. However, its association with cognitive function was less definitive, suggesting domain-specific effects. Huang et al. also found both direct and indirect associations between IADL function and cognitive status. These findings align with research on modifiable health metrics by Wang et al., where physical activity emerged as a critical factor in reducing all-cause mortality among cognitively impaired older adults, potentially averting 26.6% of deaths through ideal engagement (see Table 1 for more physical activity-related articles of this research topic).

**TABLE 1:** Physical activity and exercise-related articles in older adults

Article title	Study population	References
Functionality, muscular strength and cardiorespiratory capacity in the elderly: relationships between functional and physical tests according to sex and age	171 Spanish older adults of 72.72 ± 6.05 years (108F; 63M)	Toro-Román et al.
Physical activity improves the visual–spatial working memory of individuals with mild cognitive impairment or Alzheimer’s disease: a systematic review and network meta-analysis	34 selected articles (*n* = 3,074 participants in the meta-analysis)	Deng et al.
Effectiveness of a sensorimotor exercise program on proprioception, balance, muscle strength, functional mobility and risk of falls in older people	45 Brazilian older adults of 84.6 ± 8.4 years (33F; 12M)	Freire and Seixas
A virtually supervised exercise program improved fitness and mental wellness in healthy and comorbidity older adult individuals during the COVID-19 pandemic	44 Mexican older adults	Canton-Martínez et al.
Move for Life an intervention for inactive adults aged 50 years and older: a cluster randomised feasibility trial	601 Irish older adults of ∼63 years (483F; 118M)	Woods et al.
Effects of Baduanjin exercise on cognitive frailty, oxidative stress, and chronic inflammation in older adults with cognitive frailty: a randomized controlled trial	102 Chinese older adults over 60 years (63F; 39M)	Ye et al.
Does acute aerobic exercise enhance selective attention, working memory, and problem-solving abilities in Alzheimer’s patients? A sex-based comparative study	53 Tunisian older adults of 70.54 ± 0.88 years (30F; 23M)	Ben Ayed et al.
Machine learning approach to classifying declines of physical function and muscle strength associated with cognitive function in older women: gait characteristics based on three speeds	198 Korean older women of ≥65 years	Kim et al.
Influence of daily life and health profile in subtle cognitive decline of women residing in Spanish religious communities: DeCo religious orders study	322 Spanish older women of ≥75 years	Lopez de Coca et al.
Relationship between domain-specific physical activity and cognitive function in older adults – findings from NHANES 2011–2014	2924 U.S. older adults of 69.20 ± 6.65 years (1504F; 1420M)	Wu et al.
The effect of juggling on the proprioceptive and attentional abilities among older women	20 Polish older women (69.95 ± 4.58)	Malik et al.
Dual group-based trajectories of physical activity and cognitive function in aged over 55: a nationally representative cohort study	5,765 Chinese adults over 55 years old	Wang et al.
Prevalence of falls and associations with family functioning among community-dwelling older adults in Guangzhou, China	2,399 Chinese older adults of >65 years (1370F; 1029M)	Sun et al.
The examination of physical function and cognitive outcomes in middle-to-older high-risk adults: an unsupervised clustering method	215 U.S. adults of 59.7 ± 14.1 years (158F; 57M)	Gills et al.

Among older adults with cognitive impairment, adherence to ideal health metrics (e.g., ≥150 min/week of physical activity, Healthy Eating Index ≥60) reduced all-cause mortality risk by 48% and 63%, respectively, as demonstrated by Wang et al. Notably, smoking cessation was associated with an 85% reduction in cancer-related deaths, emphasizing the life-saving potential of lifestyle modifications even after cognitive decline begins. In addition, Kawabata et al. demonstrated the feasibility of a darts game intervention in older adults with mild cognitive impairment and highlighted the results of the center of gravity shift test as indicative of early cognitive function decline. Hao and Kim reported a positive association between participation in leisure activities and cognitive function, underscoring the potential cognitive benefits of leisure involvement for older adults with disabilities.

## Heterogeneity in cognitive trajectories and predictors

Shen et al. examined the physical aging characteristics of the older people over 70 years old in China, highlighting declines in vital capacity, flexibility, muscle strength, cardiorrespiratory fitness, and balance as key features of the aging process. Notwithstanding, cognitive decline is not uniform across aging populations. A latent growth mixture model analysis of 983 disabled older adults by Pang et al. identified three distinct trajectories: *rapid decline* (32.6%), *slow decline* (36.1%), and *stable cognition* (31.2%). Key predictors of stability included younger age (45–59 years), higher education, urban residence, and social participation. Conversely, rural residency, low income, and depression were linked to rapid decline. This heterogeneity underscores the need for interventions tailored to socioeconomic and health profiles. Sex-related differences also require additional research, as Pal et al. demonstrated that being female, a previous diagnosis of atrial fibrillation, and stroke are risk factors for advanced cognitive dysfunction.

Interestingly, Huang et al. emphasized the crucial role of intergenerational family support—especially from children—in reducing loneliness among older adults. Authors adviced that governments strengthen regulations on children’s alimony support and enhance digital infrastructure to deepen and broaden family-based care for the elderly. As noted by Ai et al., there is a fundamental connection between disability and cognitive impairment in older adults, emphasizing that social relationships and depressive symptoms can directly or indirectly influence this association.

## Future directions

To translate evidence into practice, future research should prioritize.

• Personalized interventions: develop algorithms to predict individual cognitive trajectories using biomarkers, socioeconomic data, and lifestyle factors. The diagnostic potential of alternative strategies (e.g., Traditional Chinese medicine) (Wang et al.) and genotype interactions (Li et al.) deserve further research.
• Longitudinal mechanistic studies: Yang et al. showed a positive non-linear relationship between the albumin-to-globulin ratio and cognitive function among U.S. older adults using 2011–2014 NHANES data. Also, there is evidence that physical exercise improves vascular health, its neuroprotective effects may be mediated by inflammation reduction or neurogenesis. In fact, acute cardiorrespiratory exercise likely enhances cerebral blood flow and BDNF secretion, offering transient but actionable cognitive benefits even in diagnosed Alzheimer’s patients (Gao et al.). Clarifying the temporal mechanistic features of sarcopenia (Cannataro et al., 2021) and the relationship between physical activity, depression, social isolation, and cognitive decline is an area of ongoing research.
• Development of integrative programs: beyond physical activity, composite health behaviors—such as diet quality, smoking cessation, and sleep hygiene—play pivotal roles in longevity. This is relevant considering the association between multimorbidity and the risk of cognitive decline (Zhang et al.). Future studies should contribute to the evaluation and standardization of combining physical activity–specially multimodal exercise (de Rondao et al., 2023), and cognitive training with nutritional strategies (e.g., high-protein diet) and optimal sleep hygiene, as seen in successful models for reducing depressive symptoms and mortality. Also, as highlighted by Bonilla et al., the power of creatine monohydrate supplementation plus resistance training should be noticed considering its robust safety profile and benefits in older populations (Xu et al., 2024; Kreider et al., 2025; EFSA Panel on Dietetic Products NaA, 2016).
•Policy advocacy, address structural determinants (e.g., income inequality, rural healthcare deserts) through subsidies for healthy aging programs and caregiver support initiatives (Izquierdo et al., 2021). Rural populations and those with limited education face compounded risks, highlighting the need for equity-focused public health strategies (Liu et al.). Furthermore, as demonstrated by Li and Liu, better spatial accessibility to community health services may enhance the ability of older adults to perform activities of daily living. Finally, Wei et al. reported that both observational and causal evidence support the link between socioeconomic position and sensory impairments, suggesting that early detection, targeted interventions, and educational efforts (Zhang et al.) can help prevent these conditions in middle-aged and older adults.

Physical function and cognitive health in aging is shaped by a complex matrix of modifiable and non-modifiable factors. The evidence underscores the viability of lifestyle interventions—particularly physical activity, nutrition, and sleep (Bonilla et al., 2024) as well as social engagement, and smoking cessation—as cost-effective strategies to delay decline and reduce mortality. By adopting a multidimensional framework that integrates individual, community, and policy-level efforts, we can foster resilience in aging populations and alleviate the global burden of cognitive impairment.
